# Modeling the Matrix-Cilium-Golgi continuum in hyaline chondrocytes by electron tomography

**DOI:** 10.1186/2046-2530-1-S1-P39

**Published:** 2012-11-16

**Authors:** T Poole, M Jennings, RJ Walker

**Affiliations:** 1 University of Otago, New Zealeand

## 

The two dimensional ultrastructure of connective tissue primary cilia shows a physical continuum between the biomechanically functional extracellular matrix, the mechanosensory primary cilium, and the polarized Golgi apparatus required to secrete an extracellular matrix. Our objective was to create an ultrastructurally accurate, tomographic model of the Matrix - Cilium - Golgi - Continuum using optimally fixed chick embryo sternal chondrocytes as a model connective tissue. Proteoglycan granules consisted of multiple minor aggrecan precipitates interconnected three-dimensionally by collagen fibers and linked directly to the ciliary membrane. These receptor-mediated attachments were matched by protein complexes linking the ciliary membrane to axonemal microtubular doublets, each coated with numerous microtubule-associated proteins. Doublets varied in length and position, and all were twisted or bent relative to the basal centriole. Transitional fibers linked the distal end of the basal centriole to the membrane junction between cell and ciliary membranes, while basal feet proteins spanned three triplets of the basal centriole and anchored microtubules radiating from the centrosome. The proximal centriole lacked appendages, but formed protein linkages with nuclear pores. The cis, medial and trans Golgi compartments were polarised by the orientation of the cilium. We have developed an integrated, anatomically accurate, tomographic model of the matrix-cilium-Golgi continuum in chondrocytes that provides a new interactive tool to examine the structural relationship between primary cilia, the ciliary proteome and matrix biomechanics. It will be used to investigate the functional relationship between the mechanosensory primary cilium of connective tissue cells and the mechanically responsive extracellular matrix they produce.

